# Effect of three different needle holders on gastrointestinal anastomosis construction time and bursting pressure in equine jejunal segments

**DOI:** 10.1186/s12917-021-02871-4

**Published:** 2021-04-15

**Authors:** Kate Averay, Gaby van Galen, Michael Ward, Denis Verwilghen

**Affiliations:** 1 Camden Equine Centre, Sydney School of Veterinary Science, University of Sydney, 410 Werombi Rd, Brownlow Hill, Sydney, New South Wales 2570 Australia; 2 Sydney School of Veterinary Science, University of Sydney, 410 Werombi Rd, Brownlow Hill, 2570 New South Wales Australia

**Keywords:** Horse, Surgical instruments, Anastomosis, Operative time

## Abstract

**Background:**

Equine small intestinal resection and anastomosis is a procedure where optimizing speed, without compromising integrity, is advantageous. There are a range of different needle holders available, but little is published on the impact surgical instrumentation has on surgical technique in veterinary medicine.

The objectives of this study were to investigate if the needle holder type influences the anastomosis construction time, the anastomosis bursting pressure and whether the bursting pressure is influenced by the anastomosis construction time.

Single layer end-to-end jejunojejunal anastomoses were performed on jejunal segments harvested from equine cadavers. These segments were randomly allocated to four groups. Three groups based on the needle holder type that was used: 16.5 cm Frimand (Group 1), 16 cm Mayo-Hegar (Group 2) or 20.5 cm Mayo-Hegar (Group 3) needle holders. One (Group 4) as control without anastomoses. Anastomosis construction time was recorded. Bursting pressure was determined by pumping green coloured fluid progressively into the lumen whilst recording intraluminal pressures. Maximum pressure reached prior to failure was recorded as bursting pressure. Construction times and bursting pressures were compared between needle holder, and the correlation between bursting pressure and construction time was estimated.

**Results:**

Construction times were not statistically different between groups (*P* = 0.784). Segments from Group 2 and Group 3 burst at a statistically significantly lower pressure than those from Group 4; *P* = 0.031 and *P* = 0.001 respectively. Group 4 and Group 1 were not different (*P* = 0.125). The mean bursting pressure was highest in Group 4 (189 ± 61.9 mmHg), followed by Group 1 (166 ± 31 mmHg) and Group 2 (156 ± 42 mmHg), with Group 3 (139 ± 34 mmHg) having the lowest mean bursting pressure. Anastomosis construction time and bursting pressure were not correlated (*P* = 0.792).

**Conclusions:**

The tested needle holders had a significant effect on bursting pressure, but not on anastomosis construction time. In an experimental setting, the Frimand needle holder produced anastomoses with higher bursting pressures. Further studies are required to determine clinical implications.

**Supplementary Information:**

The online version contains supplementary material available at 10.1186/s12917-021-02871-4.

## Background

Small intestinal resection and anastomosis has been estimated to be required in 22% [1] to 25% [2] of equine surgical colic cases. The reported short term survival rates for horses requiring small intestinal resection and anastomosis vary from 76 to 88% [1, 3–7], and are lower than reported survival of horses with small intestinal lesions not requiring anastomosis; 82.7% [2] to 89% [3], with a higher incidence of complications [3, 7]. In emergency equine exploratory laparotomies, duration of surgery is directly related to risk of surgical site infections (SSI) and inversely related to the survival rate [8, 9]. One equine study demonstrated that horses with greater than 180 min exploratory laparotomy surgical time had a 3.5 times greater risk of non-survival [1]. Another study demonstrated the incidence of incisional complications was 24% amongst horses with less than 2 h exploratory laparotomy surgical time, and 47% amongst horses with more than 2 h surgical time in the same institution [10]. A small animal orthopaedic surgery study estimated a 2% increase in risk of SSI for each minute of anaesthetic time [11]. Even though complexity of the gastrointestinal pathology is obviously a bias for the duration of surgery, optimizing surgical speed in horses undergoing exploratory laparotomy is still generally accepted to be of importance. Improving speed of gastrointestinal anastomosis execution, without compromising the integrity of the surgical procedure, may therefore contribute to reducing the overall duration of exploratory laparotomy. The anastomosis technique used can influence the resultant lumen diameter [7, 12, 13] as well as the intestine’s ability to withstand peristaltic movement and intraluminal pressure from ingesta [13–15]. Leakage or stenosis at the anastomosis site, manifesting clinically as postoperative ileus or a functional obstruction [1, 4], recurrent colic [1, 4, 7] and septic peritonitis [1, 4, 7] are feared complications.

As the field of surgical techniques develops, there is increasing consideration of the impact of instrument design [16]. Recognition that modern surgeons are diverse in height, hand size and gender has led to an emphasis on providing instruments that can be utilized comfortably and effectively by surgeons of a wide range of sizes and strengths [17]. This movement is also driven by the high incidence of work-related musculoskeletal disorders amongst both human [18] and veterinary surgeons [19].

There are a range of different needle holders available for use in veterinary medicine, however, the majority of designs are based on the Mayo-Hegar needle holder [20]. Most needle holders are available in a range of sizes; size selection is based on intended suturing task, size of suture being used and surgeon preference. Whilst there is little published on the impact of design or size of needle holders on surgical technique, the Frimand needle holder has been shown to reduce suturing time and surgical stress in an experimental setting [20]. There is currently no data on its performance in anastomosis construction in horses with colic.

The objectives of this study were to investigate if 1.) the needle holder type influences the speed of construction (“anastomosis construction time”), 2.) the needle holder type influences the intraluminal pressure the anastomosed segment withstood before leaking (“bursting pressure”) and 3.) the “bursting pressure” is influenced by the “anastomosis construction time”.

We hypothesized that the type of needle holder would impact anastomosis construction time but would not influence anastomosis bursting pressure. We also hypothesized that the bursting pressure would not be correlated with anastomosis construction time in this study.

## Results

### Description of Jejunal segments

All jejunal segments were macroscopically determined normal and without pathology. The jejunal segments from each cadaver were randomly allocated into and equally distributed amongst the 4 groups (Tables 1 and 2). A total of 46 anastomoses were performed. The anastomoses were performed over 12 different sessions, with the number of anastomoses per session ranging from 1 to 6 (median 3.5 anastomoses).

**Table 1:** The construction times and times per suture of end-to-end jejunojejunal anastomosis performed on equine cadavers

	Number of anastomoses	Anastomosis Construction times (sec)				Number of sutures per anastomosis				Time per Suture (sec)			
	*n*	Min	Mean	Max	St Dev	Min	Mean	Max	St Dev	Min	Mean	Max	St Dev
**All Anastomoses:**	**46**	**538**	**723**	**894**	**90**	**16**	**20**	**25**	**1.8**	**31**	**36**	**41**	**2.9**
(Group 1) FR	15	631	736	894	78	16	20	25	2.3	33	36	41	2.3
(Group 2) SMH	15	582	715	846	83	17	20	23	1.4	32	35	41	2.9
(Group 3) LMH	16	538	717	880	110	17	19	22	1.6	31	36	41	3.5
(Group 4) Control	34	–	–	–	–	–	–	–	–	–	–	–	–

Legend: *n* Sample size, *Min* Minimum, *Max* Maximum, *St Dev* Standard Deviation, *LMH* 20.5 cm Mayo-Hegar Needle Holder, *SMH* 16 cm Mayo-Hegar Needle Holder, *FR* 15 cm Frimand Duo-Grip Needle Holders

**Table 2:** The bursting pressures of end-to-end jejuno-jejunal anastomosis performed on cadaveric samples

	Number of Anastomoses	Bursting pressure (mmHg)			
	*n*	Min	Mean	Max	St Dev
**All Anastomoses:**	**80**	**84.0**	**168.6**	**286.0**	**51.9**
(Group 1) FR	15	120	166	208.0	31
(Group 2) SMH^*a*^	15	84	156	232.0	42
(Group 3) LMH^*b*^	16	86	139	208.0	34
(Group 4) Control^*c*^	34	84	189	286	61.9

Legend: *n* Sample size, *Min* Minimum, *Max* Maximum, *St Dev* Standard Deviation, *LMH* 20.5 cm Mayo-Hegar Needle Holder, *SMH* 16 cm Mayo-Hegar Needle Holder, *FR* 15 cm Frimand Duo-Grip Needle Holders. ^*b-c*^ = **, ^*a-c*^ = *

The time between euthanasia of cadaver and bursting of jejunal segments ranged from 3.8 h to 47.9 h, with a median time of 21.8 h.

### Anastomosis construction time

Anastomosis construction times are reported in Table 1. Anastomosis construction time was normally distributed. The mean anastomosis construction time was shortest with SMH followed by the LMH, with the FR having the longest mean construction time but showing more consistency in construction times (lower standard deviation). However, the anastomosis times were not statistically different between needle holders (*P* = 0.784).

The time per suture is reported in Table 1. The range and mean values were similar across the three needle holders, but the standard deviation was lowest for FR (therefore showing the highest consistency in time per suture) followed by SMH and LMH. The time per suture did not significantly differ between needle holders (*P* = 0.479).

### Bursting pressure

The anastomosed segments burst either at the mesenteric edge of the anastomosis (37%, *n* = 17/46), at multiple sites along the anastomosis (45.7%, *n* = 21/46) or into the mesentery at a site distant to the anastomosis (17.4%, *n* = 8/46). The majority of un-anastomosed segments/controls (94.1%, *n* = 32/34) burst into the mesentery; the remaining two segments burst at the point where attached to the manometer. Bursting pressures are reported in Table 2. The mean bursting pressure was highest in Group 4 (no anastomosis), followed by Group 1 (FR) and Group 2 (SMH) with Group 3 (LMH) having the lowest mean bursting pressure. Bursting pressure was normally distributed.

The bursting pressure was statistically different between the groups; both Group 2 (SMH) and Group 3 (LMH) segments burst at a significantly lower pressure than Group 4 (no anastomosis) segments; *P* = 0.031 and *P* = 0.001 respectively. There was no significant difference between the bursting pressures of Group 1 (FR) and Group 4 (no anastomosis) segments (*P* = 0.125). These differences were not confounded by cadaver nor session.

### Anastomosis construction time and bursting pressure

There was no significant association between anastomosis construction time and bursting pressure (Pearson correlation 0.04, *P* = 0.792).

## Discussion

The hypothesis of this study was that the needle holder type would influence the time taken to construct an end-to-end jejunojejunal anastomosis in an equine cadaver model. Our hypothesis was rejected; there was no significant difference (*P* = 0.784) in anastomosis construction times between the three needle holders tested. The FR needle holder, though, showed more consistency in construction times and suture times (smallest standard deviation). Furthermore, the overall mean anastomosis construction time of 12.0 ± 1.5 min (723 ± 90 s), was faster than other published hand-sewn single layer end-to-end anastomosis construction times; 21.10 ± 3.29 min [14], 19.52 ± 1.40 min [21] and 15.6 ± 0.72 min [22]. The needle holder used was not specified in these three studies [14, 21, 22]. The faster anastomosis time in this study may reflect the experience of the surgeon; the surgeons in the three other studies were specified to be either a resident [21], a non-boarded clinician [22] or indicated that each anastomosis technique was practiced 20 times prior to the study [14]. In contrast to the findings of Frimand Rönnow and Jeppsson [20], use of the Frimand needle holder did not reduce suturing time in the current study, when compared to the Mayo-Hegar needle holders.

Including biomechanical testing in this study design was intended as a deterrent for sacrificing surgical precision for surgical speed. Yet, anastomosis construction time did not influence the bursting pressure of anastomosed jejunal segments. The control jejunal segments had similar bursting pressures, 189 ± 62 mmHg, to control segments in other studies using biomechanical testing with water (200.41 ± 14.17 mmHg [23], 227 ± 20.3 mmHg [15] and 257.63 ± 45.41 mmHg [24]); but bursting pressures in the current study were lower than those reported when biomechanical testing was performed with air, (290.6 ± 35.06 mmHg [13] and 397.6 ± 103.4 mmHg [25]). The bursting pressure of all anastomosed segments (169 ± 52 mmHg), were also similar to the bursting pressures of other hand-sewn single layer anastomoses in other studies (134.5 ± 73.05 mmHg [14], 208 ± 18.2 mmHg [15], 253.81 ± 23.79 mmHg [21], 251.56 ± 56.79 mmHg [13]), and greater than the bursting pressures of stapled end-to-end anastomoses (48.64 ± 14.17 mmHg [23] and 67.05 ± 20.79 mmHg [21]). Interestingly the bursting pressure of the control segments showed a wide variability, with the lowest value as low as the lowest value of anastomosed gut, without any confounding between cadavers. The large standard deviation of the bursting pressure measurements seen in our study could be attributed to the inherent variability during the anastomoses construct and to outliers of our data set.

An unexpected finding of the present study was that the needle holder used had an impact on the bursting pressure of anastomoses. The bursting pressures of anastomoses performed with the LMH and SMH needle holders were significantly lower than the control segments. Anastomoses performed with the FR needle holder burst at a higher pressure than the LMH and SMH anastomoses and were no different in bursting pressure to control segments.

The impact of needle holder on anastomosis bursting pressure might reflect the friable nature of gastrointestinal tissue and the potential relationship between needle holder design and exerted force on tissue when suturing. Macroscopic damage is seen in porcine small intestine following application of a mean of 218 g (~ 2 N) traction force and rupture will occur at a mean of 300 g (~ 3 N) [26]. During suturing, the amount and velocity of hand movements impacts the force placed on tissue [27]. Measured forces applied to tissue during real and stimulated suturing models vary from 0.5–3.5 N [27–29]. Iatrogenic tissue trauma whilst suturing can be minimized by introducing the needle perpendicular to the tissue surface and following the curvature of the needle [28]. Uncontrolled movement when handling the needle - such as needle wavering (redirecting the needle mid-bite) to increase accuracy [30] or quivering when disengaging the ratchet to release the needle [20, 31] - can result in tissue trauma [30]. The Frimand needle holders are designed to allow smooth disengagement of the ratchet and have been shown to result in less than 158 g (~ 1.5 N) of force during suturing [20]; this suggests that less tissue trauma may have occurred during anastomoses with the Frimand needle holders. Less iatrogenic trauma during the anastomosis may have resulted in the higher bursting pressures seen in this study.

The bursting pressures seen in all tested groups in this study are greater than can be expected in clinical cases. During intestinal obstructions, degenerative changes, starting with the development of oedema in the lamina propria of the equine jejunal wall, are seen with intraluminal pressures as low as 13.2 mmHg [14, 32]. In a study comparing the intra-luminal pressures of horses with small intestinal obstructions, the mean intra-luminal pressure of non-survivors was 11 mmHg [33]. The anastomoses in this study withstood substantially greater intra-luminal pressures than have been demonstrated clinically. However, a study in mice has demonstrated that the strength of anastomosed jejunal segments reduces to 15% of the immediate post-operative strength over the first 3 days following surgery, before increasing in strength again [34]. Fifteen percent of the mean bursting pressure of all anastomosed segments in the present study would equate to 25 mmHg (26 mmHg for Frimand, 20–22 mmHg for the Mayo-Hegars needle holders), which is not that much greater than reported pressures seen in clinical cases. If such weakening of the anastomoses does occur in equine patients, obtaining increased anastomosis strength by only a few mmHg’s due to the use of a different type of needle holder may well have significant clinical relevance to the actual construct and the potential to avoid post-operative anastomosis leakage or rupture.

There are a wide range of different techniques used for end-to-end jejunojejunal anastomoses [14, 23, 24]. Traditionally a two-layer closure has been used, but single layer anastomoses have been shown to be faster [14], biomechanically equivalent [24] and clinically comparable [12, 35]. Both stapled and hand-sewn techniques, including a range of inverting and apposing suture patterns, are used [4]. Whilst there is concern that interrupted suture patterns increase the risk of adhesions due to exposure of suture material [36], decreasing the time taken and amount of manipulation required to construct the anastomosis is believed to reduce mural inflammation and reduce risk of both adhesions and post-operative ileus [7, 37]. Use of a single layer interrupted suture pattern has been shown to be a safe and effective technique [38].

One study limitation is the use of cadaver material. Therefore this study does not account for the impact that in vivo factors, such as haemorrhage, oedema, development of a fibrinous seal and perfusion of the anastomosis edges, have on anastomosis technique and post-operative healing [39]. Using cadaver material also introduces the risk that tissue autolysis may impact the integrity of jejunal segment [39]. However, there was no confounding effect of the cadaver on the findings. All jejunal segments were chilled, either in ice water or in a refrigerator, until use. A previous study has shown that there is no difference in maximum intra-luminal bursting pressure between fresh, chilled and frozen then thawed jejunal segments [39].

The biomechanical testing model has been used in many other studies comparing the strength of ex vivo anastomoses [13–15, 22–25]. Several of these studies have calculated and used bursting wall tension (BWT) rather than bursting pressure alone. BWT has been coined as superior to bursting pressure, as it accounts for longitudinal and circumferential forces in a distensible tube [23]. However, calculation of BWT requires a fixed length of intestinal segment [15]. Whilst jejunal segments were measured and secured into the bursting model at a fixed length, the intestinal length increases as the jejunal segment is filled with water and expands during the testing. This has been suggested to be similar to the physiological response to increased intra-luminal pressures [25], but the dynamic nature of the segment length during the testing procedure may render calculations of actual BWT less accurate. Therefore, bursting pressure alone was used in this study.

Another limitation is that only one surgeon was used in this study. Whilst this was done to minimize random error, the findings in this study might be affected by the surgeon’s individual technique [40] and experience. This surgeon had more than 15 years of experience with Mayo-Hegar needle holders of varying sizes, and prior to this study had not used Frimand needle holders. The surgeon could not be blinded to the needle holder type. Enrolling multiple surgeons and using a needle holder that is novel to each surgeon for comparison may reduce the bias of surgeon preference in future studies.

## Conclusion

In conclusion, this study suggests that the needle holder type affects anastomosis bursting pressure but does not affect anastomosis construction time. The FR needle holder, with sutured intestinal segments that burst at a similar pressure than none-anastomosed control segments, performed better than the Mayo-Hegar needle holders of two different sizes. It also showed the highest consistency in suture times and bursting pressures. Therefore, the FR needle holder shows promise to be superior for anastomoses in a clinical setting. However, future research is needed to confirm the clinical significance of this finding.

## Methods

### Sample size calculations

Sample size calculations were performed assuming a mean anastomosis construction time of 20 min with a standard deviation of 1 min, based on previous studies, [13, 14, 24] an expected 10% decrease in anastomosis time, 90% power and a 0.05 alpha (https://clincalc.com/stats/samplesize.aspx). Calculations determined a minimum of 5 anastomosis per group were required.

### Collection of Jejunal specimens

Jejunal segments were collected from 6 horses (3 females, 3 males; 3 standardbreds, 2 thoroughbreds, 1 mixed breed), with estimated weights ranging from 400 to 500 kg (483 kg ± 41 kg) and reported ages ranged from 2 years to 17 years (5.2 years ±5.9 years), slaughtered at commercial abattoirs using captive bolt.

Within 45 min of euthanasia, the gastrointestinal tract was examined to confirm there were no gross abnormalities present. The jejunum was then harvested; ingesta was milked orally to aborally to the transected ileum and evacuated. Jejunal segments were submerged into ice water for transport and stored at 4 °C until utilized.

The jejunum was divided into 40 cm segments. Segments were labelled consecutively from oral to aboral, and randomly allocated into four groups: anastomosis with 15 cm Frimand Duo-Grip (FR) needle holders (Group 1), anastomosis with 16 cm Mayo-Hegar (SMH) needle holders (Group 2), anastomosis with 20.5 cm Mayo-Hegar (LMH) needle holders (Group 3); or control Group (no anastomosis performed; Group 4).

### Anastomosis construction

**Fig. 1:**
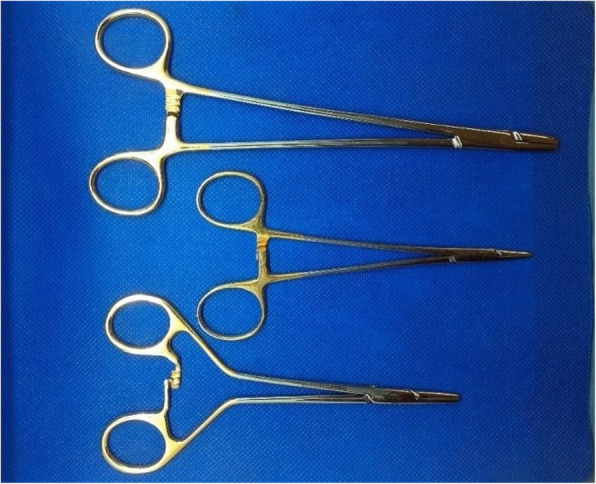
The needle holders used for end-to-end single layer interrupted Lembert jejunojejunal anastomoses in equine cadavers. Legend: Stille 20.5 cm Mayo-Hegar Needle Holders (LMH), Middle: Stille 16 cm Mayo-Hegar Needle Holders (SMH), Bottom: Stille 15 cm Frimand Duo-Grip Needle Holders (FR)

Single layer end-to-end jejunojejunal anastomoses were performed on the Group 1, 2 and 3 segments using a simple interrupted Lembert pattern as described elsewhere [41]. A 3–0 monofilament absorbable suture (72% glyconate; Monosyn, B. Braun Surgical, Rubi, Spain) on a 26 mm taper needle was used by the same right-handed ECVS Diplomate with each of three randomly allocated needle holders (Fig. 1):

1. 15 cm Frimand duo-grip (FR; Stille surgical instruments, Torshälla, Sweden)
2. Stille 16 cm Mayo-Hegar (SMH; Stille surgical instruments, Torshälla, Sweden) and
3. Stille 20.5 cm Mayo-Hegar (LMH; Stille surgical instruments, Torshälla, Sweden)

Jejunal segments were sharply transected using Metzenbaum scissors. Stay sutures were placed at the mesenteric and antimesenteric borders of each portion of transected jejunal segment to allow the assistant to apply tension to appose the jejunal portions as required during the anastomosis. Suture bites were taken approximately 2–3 mm from the incised edge. An Adson Brown tissue forceps was used to manipulate tissue and suture needle in a no touch technique. The assistant cut suture tails as directed by the surgeon.

Each anastomosis construction was timed and videoed for later review. The time from first contact of needle with jejunum to the suture tail of last suture being cut was recorded as “anastomosis construction time”. The number of sutures placed were recorded for each anastomosis, and the anastomosis construction time was divided by the number of sutures placed to calculate the “time per suture”. The cadaver that the jejunal segment originated from and the session in which the anastomosis was performed were considered to be potential confounders, so were also recorded.

### Biomechanical testing

**Fig. 2:**
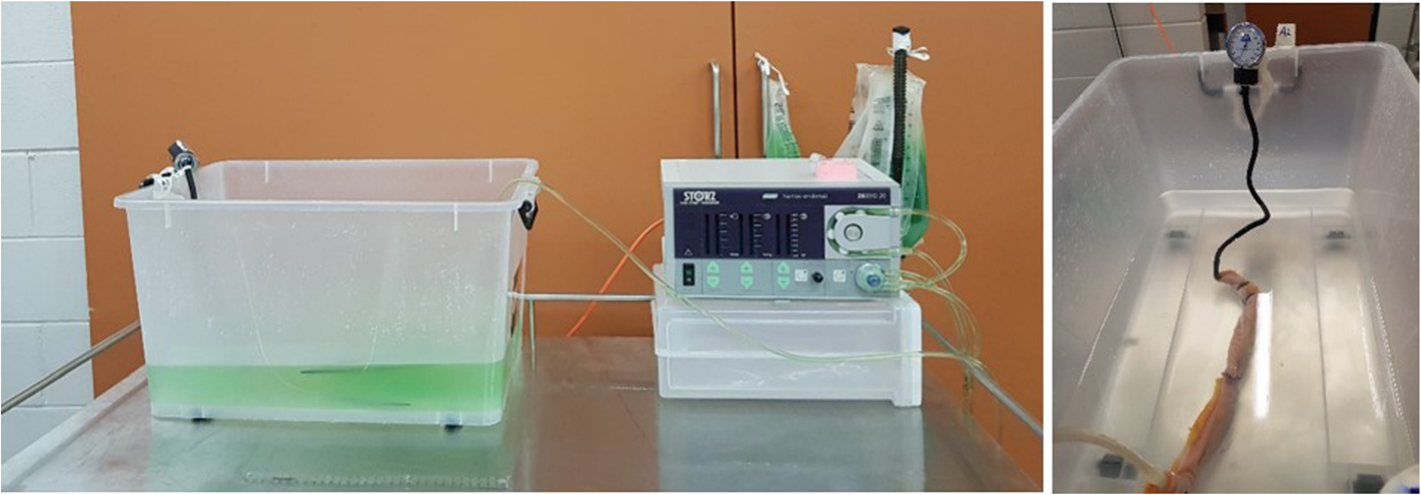
The experimental set up to test bursting pressure in end-to-end jejunojejunal anastomoses from equine cadavers. Legend: An irrigation pump with hung fluids coloured with green food dye was connected to one end of the jejunal segment and secured with a zip tie. The other end of the jejunal segment was connected to a blind-ended pressure manometer. The green coloured fluid was progressively pumped into the jejunal lumen whilst intraluminal pressures were recorded until the anastomosis or intestinal segment failed

Bursting pressures of group 1, 2, 3 and 4 segments were determined using a previously described technique [14, 15, 22]. Immediately after the anastomosis was performed, the jejunal segments were individually submerged in a bath of room temperature water. An irrigation pump (Karl Storz, Hamou Endomat 26,331,120) with fluids coloured with green food dye (Queen, Green Food Colouring) was connected to one end of the jejunal segment and secured with a zip tie at 15 cm from the anastomosis, with 5 cm excess jejunum left on the non-pressurized side of the zip tie. The other end of the jejunal segment was connected to a blind-ended pressure manometer (Livingstone, Aneroid blood pressure sphygmomanometer), again 15 cm from the anastomosis with 5 cm excess jejunum external to the zip tie (Fig. 2). Control segments were secured with 30 cm segments between the connection for the fluids and manometer, leaving 5 cm excess jejunum either side of the pressurized segment.

The green coloured fluid was progressively pumped into the jejunal lumen (with the irrigation pump set to maximum pressure of 400 mmHg and flow rate 100 ml/min) whilst intraluminal pressures were recorded until the anastomosis or intestinal segment failed. Maximum pressure reached prior to failure was recorded as “bursting pressure” in mmHg and the location of egressing fluid at that point was recorded.

### Statistical analysis

Descriptive statistics for the cadavers, jejunal segments, number of anastomoses in each group, number of sutures placed and time between euthanasia and testing were calculated and reported as mean ± standard deviation (SD).

Normality of the anastomosis construction time and bursting pressure was determined using Kolmogorov-Smirnov and Shapiro-Wilk tests. Mean anastomosis construction time and bursting pressure were compared between the 3 groups using generalized linear models and then recalculated with additional variables added as random effects. Comparison of the marginal means of the adjusted and unadjusted models were used to identify confounders; confounding was confirmed if the adjusted model marginal means varied from the unadjusted model by > 20%.

Pearson’s correlation was used to determine correlation between anastomosis construction time and bursting pressure.

Statistical analyses were performed using R studio software (RStudio Version 1.2.5001, Boston, MA) and SPSS (IBM Corp. Released 2016. IBM SPSS Statistics for Windows, Version 24.0. Armonk, NY: IBM Corp.). *P* < 0.05 was considered significant for all statistical tests.

## Supplementary Information

**Additional file 1.** Construction time and bursting pressure in jejunal anastomosis. Raw data collected in this study with legend.

## Data Availability

The dataset supporting the conclusions of this article is included as an excel document.

## Abbreviations

FR: 15 cm Frimand Duo-Grip Needle Holders
SMH: Stille 16 cm Mayo-Hegar Needle Holders
LMH: Stille 20.5 cm Mayo-Hegar
